# Patient perspectives on virtual consultations for type 2 diabetes in the United Kingdom: use, associated factors, and perceived quality of care

**DOI:** 10.3389/fdgth.2026.1921014

**Published:** 2026-10-12

**Authors:** Reham Aldakhil, Geva Greenfield, Albandari Alharbi, Ahmed Alboksmaty, Benedict Hayhoe, Azeem Majeed, Ana Luisa Neves

**Affiliations:** 1 Department of Primary Care and Public Health, Imperial College London, London, United Kingdom; 2 Institute of Global Health Innovation (IGHI), Department of Surgery and Cancer, Imperial College London, London, United Kingdom

**Keywords:** digital health literacy, health equity, technology adaptation, type 2 diabetes, virtual consultation

## Abstract

**Introduction:**

Although virtual consultations (VCs) have become a routine component of type 2 diabetes (T2D) care, evidence regarding patient experiences remains limited. We investigated VC use among UK adults with T2D, factors associated with both current and intended use, and perceived quality of care.

**Methods:**

A weighted national cross-sectional survey of 373 UK adults with self-reported T2D was conducted via the YouGov panel. The questionnaire included digital adoption factors based on the UTAUT2 framework, and perceived impacts of virtual consultations across IOM quality domains. Weighted logistic regression identified predictors of use and satisfaction.

**Results:**

About one in three respondents (31.0%) had used virtual consultations, almost always by telephone (91.3%) and infrequently. After adjustment, younger age (18–44 vs. ≥65: OR 7.76, 95% CI 2.22–27.16) and a bachelor's undergraduate education (OR 2.05, 95% CI 1.06–3.96) were the only independent factors associated with use, and the same profile predicted intention to use. In the acceptance framework UTAUT2, effort expectancy and facilitating conditions were the strongest constructs and social influence the weakest. Across the full sample, efficiency was perceived as the quality domain most positively impacted by virtual consultations (62.4%), whereas patient-centredness was rated least favourably (34.5%). Perceived equity was also limited, with only 40.2% of respondents agreeing that different groups benefit equally from virtual consultations. Users rated the five parallel quality domains more positively than non-users anticipated; the equity item, asked of all respondents, did not differ significantly between groups.

**Conclusion:**

Findings suggest that equitable and sustained uptake of virtual consultations requires attention to both digital inclusion and usability. Policies should support tailored interventions for populations at risk of exclusion, alongside investment in digital infrastructure and support resources. As perceived benefits vary across dimensions of care quality, virtual consultations are likely to be most effective when integrated within flexible hybrid models that align with patient preferences and care needs.

## Introduction

Type 2 diabetes (T2D) is one of the most common long-term conditions in the United Kingdom. More than five million people are now living with diabetes, of whom approximately 90% have T2D, and a further 1.3 million are estimated to be living with undiagnosed T2D (1). Prevalence is rising and is consistently higher among older adults, in more socioeconomically deprived communities, and in people of South Asian and Black ethnicity (2). High-quality, continuous, and accessible care is essential for effective diabetes management and prevention of complications.

Healthcare systems globally have increasingly adopted digital health solutions to support chronic disease management and improve access to care. Virtual consultations (VCs), including telephone and video consultations, have become a core component of routine healthcare delivery, particularly since the COVID-19 pandemic. Although telephone consultations were long established in UK general practice, video and online consultations were rare before 2020 (3). The annual number of remote primary care consultations in England roughly tripled, from about 10.1 million to 32.7 million between 2019 and 2022 (4).

In T2D care, VCs offer a range of potential benefits including improved convenience, reduced travel burden, and increased service flexibility (5, 6). A recent systematic review on virtual consultations in T2D found that patient satisfaction was broadly comparable to, or better than, face-to-face care, and that glycaemic control was non-inferior, with no significant difference in HbA1c reduction between the two modalities (5, 7). This is consistent with a recent pragmatic, non-inferiority randomised trial in which teleconsultation was non-inferior to face-to-face consultation for HbA1c in adults with T2D (8). Of note, much of this evidence derives from selected trial populations and structured interventions rather than the routine, real-world virtual consultations that most patients now encounter.

Given the rapid integration of VCs into routine diabetes care, there is a need to better understand how patients use and perceive the impacts of these services, aspects that remain rarely evaluated. In particular, patient-reported insights are essential for evaluating the acceptability and quality of VC-based care delivery and can generate the evidence needed to inform equitable and effective implementation of virtual care models in T2D management.

We conducted a national cross-sectional survey of adults with Type 2 diabetes in the United Kingdom to understand patterns of virtual consultation (VC) use, patient-reported perceptions of the quality of virtual care, and the key factors driving adoption of this mode of healthcare delivery. Specific objectives included: a) to describe patterns of virtual consultation use among people with T2D, including determinants of use; b) to identify factors influencing actual use/intention of use of virtual consultations, and c) to examine patient-reported perceptions of the impact of virtual consultations on quality of care across Institute of Medicine (IOM) domains of care quality (Figure 1).

**Figure 1:**
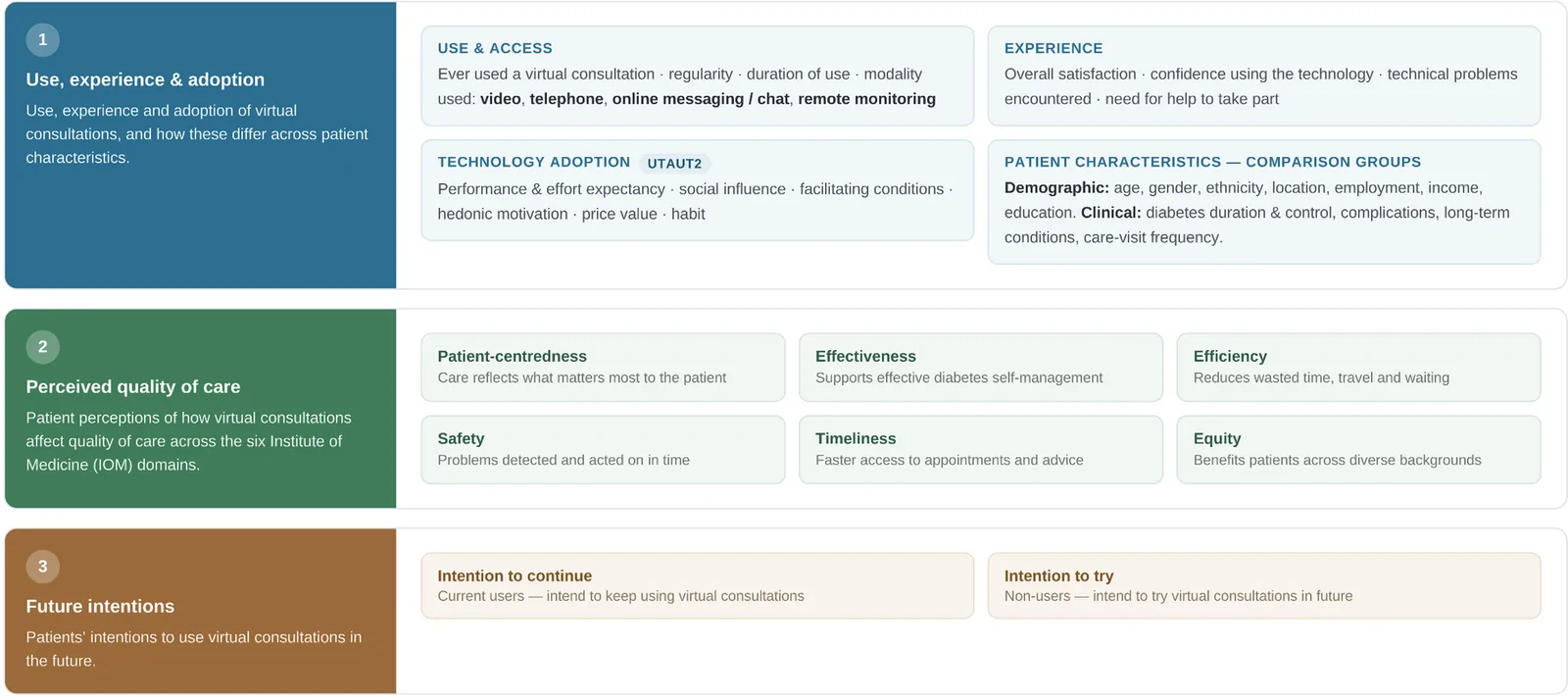
Study conceptual and analytic framework.

## Methods

### Study design and setting

A national cross-sectional online survey of UK adults with self-reported T2D was conducted between 30 January and 6 February 2026, through the YouGov UK panel (9, 10). YouGov maintains an active UK research panel of approximately 400,000 members, including more than 9,600 panel members with a recorded T2D profile from prior screening, from which the study sample was drawn. This study adhered to the CHERRIES (Checklist for Reporting Results of Internet E-Surveys) reporting guidelines for the conduct and reporting of web-based surveys (11), (Supplementary Table S6).

### Participants and recruitment

Eligible participants were UK adults aged 18 years or older who self-reported a diagnosis of T2D. Invitations were distributed to panel members profiled with T2D. Participants were recruited through the YouGov UK online panel. The survey questionnaire was designed by the research team and administered by YouGov. YouGov was responsible for participant recruitment and survey fieldwork. A total of 417 respondents started the survey, of whom 380 completed it (91.1% completion rate). Following quality screening, seven responses were excluded due to inconsistent responses or substantial missing data on key variables, resulting in a final analytic sample of *n* = 373.

### Survey instrument

A core set of demographic characteristics, age, gender, ethnicity, region, and social grade was drawn from YouGov's existing panel profiling rather than re-asked. The survey itself collected additional sociodemographic information (area type, employment status, household income, and highest educational qualification), social grade was classified using the UK National Readership Survey scheme, grouped into ABC1 (managerial, administrative and professional) and C2DE (skilled manual, semi-/unskilled and non-working). Clinical characteristics, including self-rated diabetes management, diabetes-related complications, number of long-term conditions, and frequency of diabetes-related healthcare visits were also collected. Digital health literacy was assessed using an adapted version of the 8-item eHealth Literacy Scale (eHEALS), administered on a four-point agreement scale (12, 13). Virtual consultation was defined here as telephone consultations, video consultations, online messaging or text-based chat, and remote monitoring (electronic sharing of health data). A routing question was used to classify respondents as VC users or non-users, directing subsequent survey questions accordingly (Supplementary Table S1).

Virtual consultation use (for VC users only), was assessed through questions covering frequency and duration of VC use, consultation modalities, levels of satisfaction, confidence in using technology, experiences of technical difficulties, and need for assistance during consultations. Future intention to continue or to try was also assessed, for both users and non-users, respectively.

Factors influencing behavioural intention and/or actual use of virtual consultations (for all participants), were covered using 12 items adapted from the Unified Theory of Acceptance and Use of Technology (UTAUT2) framework (14). The UTAUT2 model includes seven core constructs that explain why people adopt and use technology: Performance Expectancy, Effort Expectancy, Social Influence, Facilitating Conditions, Hedonic Motivation, Price Value, and Habit (14).

Patient-reported perceptions of the impact of virtual consultations were captured using 11 items mapped to the six IOM quality domains (15): safety, effectiveness, patient-centredness, timeliness, efficiency, and equity (16); with parallel items for users (experienced) and non-users (anticipated) for five domains, and a single equity item asked of all participants.

### Statistical analysis

All descriptive and inferential analyses applied the YouGov survey weights, so that estimates reflect the demographic profile of the UK adult population with T2D rather than the unweighted composition of the panel. Descriptive statistics summarised the sample, reporting weighted frequencies and percentages for categorical variables and design-based (survey-weighted) linear models for continuous outcomes, with Taylor-linearised standard errors. Weighted means are reported with design-based 95% confidence intervals, and because the user versus non-user comparisons were numerous and exploratory we also applied a Benjamini–Hochberg false-discovery-rate correction as a sensitivity analysis.

Determinants of ever-use of VC were modelled using weighted logistic regression: an unadjusted model for each candidate predictor [age, gender, ethnicity, education, social grade, employment, multimorbidity (≥3 long-term conditions), and eHEALS mean], followed by an adjusted multivariable logistic regression including all predictors simultaneously. The same approach was used to model predictors of satisfaction (VC users only), with eHEALS, ethnic minority status, and multimorbidity as predictors. Satisfaction was dichotomised as very or fairly satisfied versus otherwise; of 120 users, 79 (65.8%; 68.4% weighted) were satisfied, and the model was fitted on 118 complete cases with 78 events, with eHEALS entered per one-point increase. The threshold for statistical significance was *p* < 0.05. All analyses were performed in R version 4.3.0 (17).

### Missing data

Missing data was <5% for most variables. Income had the highest rate of missingness (20.6%, *n* = 77). UTAUT2 constructs had 6–17% missing due to “Don't know” responses being recoded to missing. “Don't know” responses were more common among non-users than users (for example, efficiency 16.3% vs. 4.2%; equity 40.2% vs. 23.3%), indicating they were not missing at random; item- and group-specific rates are reported in Supplementary Table S7. Analyses were complete-case, and a sensitivity analysis recoding “don't know” to the scale midpoint left all user versus non-user conclusions unchanged (12 of 13 comparisons remained significant after Benjamini–Hochberg adjustment; equity remained non-significant). A sensitivity analysis including income improved overall model fit only marginally and did not alter the main conclusions.

### Ethical approval and consent

This study was approved by the Imperial College Research Ethics Committee (ICREC; reference number 8213600) on 29 January 2026, following review by the Research Governance and Integrity Team. All participants received a participant information sheet and provided informed consent before completing the survey.

## Results

### Sample characteristics

Of the 373 participants in the sample, 120 (31.0%) reported having used a VC for their T2D care, while 251 (68.4%) had never used a VC; 0.6% preferred not to disclose their VC status. The sample was predominantly male (56.0%) and more than half of participants (55.0%) were aged 65 or older. Approximately one-third (32.2%) identified as belonging to an ethnic minority group, 49.4% had education below degree level, and 36.5% were in skilled manual, semi-/unskilled and non-working (social grade C2DE).

In terms of health status, 56.8% of the participants reported that their T2D was well managed, 27.7% reported at least one T2D-related complication, and 50.9% reported three or more long-term conditions including T2D. Participant characteristics, including the distribution of VC users and non-users, are presented in Table 1.

**Table 1:** Demographic and clinical characteristics of the sample, by ever-use of virtual consultations.

Characteristic	Total (*n* = 373)	Users (*n* = 120)	Non-users (*n* = 251)	*p*-value*
Gender				1.000
Male	209 (56.0%)	67 (55.8%)	140 (55.8%)	
Female	164 (44.0%)	53 (44.2%)	111 (44.2%)	
Age				<0.001
18–44	25 (6.7%)	16 (13.3%)	8 (3.2%)	
45–64	143 (38.3%)	47 (39.2%)	96 (38.2%)	
65+	205 (55.0%)	57 (47.5%)	147 (58.6%)	
Ethnicity				0.017
White	253 (67.8%)	71 (59.2%)	181 (72.1%)	
Ethnic minorities	120 (32.2%)	49 (40.8%)	70 (27.9%)	
Education				0.001
Below degree	176 (49.4%)	39 (35.1%)	136 (56.0%)	
Bachelor's(undergraduate)	85 (23.9%)	36 (32.4%)	49 (20.2%)	
Postgraduate or higher	95 (26.7%)	36 (32.4%)	58 (23.9%)	
Social grade				0.098
ABC1	237 (63.5%)	84 (70.0%)	152 (60.6%)	
C2DE	136 (36.5%)	36 (30.0%)	99 (39.4%)	
Employment				0.065
Working	124 (33.7%)	49 (40.8%)	75 (30.5%)	
Not working	244 (66.3%)	71 (59.2%)	171 (69.5%)	
Household income				0.007
Up to £20k	70 (23.6%)	17 (17.0%)	52 (26.7%)	
£20k–£39k	104 (35.1%)	40 (40.0%)	64 (32.8%)	
£40k–£59k	64 (21.6%)	15 (15.0%)	49 (25.1%)	
£60k+	58 (19.6%)	28 (28.0%)	30 (15.4%)	
Self-rated diabetes management				0.938
Well managed	210 (56.8%)	69 (57.5%)	140 (56.5%)	
Not well managed	160 (43.2%)	51 (42.5%)	108 (43.5%)	
Diabetes complications				0.034
Any complication	101 (27.7%)	41 (35.7%)	60 (24.3%)	
None	263 (72.3%)	74 (64.3%)	187 (75.7%)	
Long-term conditions (≥3 including T2D)	194 (50.9%)	68 (56.4%)	126 (52.3%)	0.448
Visited a provider for diabetes care (ever)	361 (96.2%)	119 (98.7%)	242 (95.8%)	0.177

* *p*-values were calculated using Rao-Scott design-adjusted *χ*^2^ tests, accounting for survey weights. Variable-specific unweighted denominators for valid responses were: education 356, employment 368, long-term conditions 361, self-rated management 370, and household income 296; all other variables 373. The two respondents who preferred not to disclose virtual-consultation status appear only in the Total column, so users + non-users = 371.

### Virtual consultation use

Among the 120 participants who had used VC for their T2D care, telephone consultations were by far the most common modality (91.3%, *n* = 110). Use of other formats was relatively uncommon, including online messaging (10.0%, *n* = 10), video consultations (7.5%, *n* = 9), and remote monitoring (7.4%, *n* = 10). Combining modalities, 74.2% of users (*n* = 90) reported telephone consultations only, whereas 21.2% (*n* = 25) reported any data-enabled modality (video, online messaging, or remote monitoring); the internet-based modalities were individually too infrequent for separate comparison. Although many participants had experience with VC over an extended period, usage frequency was generally low. Nearly half (44.6%, *n* = 54) reported using VCs for more than two years, yet 61.2% (*n* = 73) engaged with them only once a year or less, and just 4.3% used it at least monthly. Additionally, 22.8% (*n* = 27) of users had had only one or two virtual consultations in total.

Overall, users reported few barriers and largely positive experiences. Most were very or fairly confident in using the required technology (74.3%, *n* = 89), while 82.8% (*n* = 99) reported no technical problems, and 87.0% (*n* = 104) did not require assistance to join their consultation. Satisfaction was generally high, with 68.4% satisfied or very satisfied, with 22.9% neutral and 8.7% dissatisfied. Intention to continue using VC was also strong, with 69.2% (*n* = 83) stating that they were likely to use this mode of care in the future. Among non-users, 37.1% of those expressing a view (about 31% of all non-users) reported that they were likely to try virtual consultations in future.

In adjusted analyses, digital health literacy, as measured by eHEALS, was the only independent predictor of satisfaction with VC (adjusted OR 2.37, 95% CI 1.09–5.15; *p* = 0.030). No demographic characteristics were independently associated with satisfaction. The full profile of VC use and user experiences is presented in Table 2.

**Table 2:** Virtual consultation usage profile (users only, *n* = 120).

Dimension	Measure	Users (*n* = 120)
Modality	Telephone	91.3%
	Online messaging	10.0%
	Video	7.5%
	Remote monitoring	7.4%
Duration of use	>2 years	44.6%
	1–2 years	25.5%
	<1 year	12.4%
Frequency	Monthly or more often	4.3%
	A few times a year/ about yearly	56.9%
	Less than once a year	10.0%
	Only 1-2 consultations ever	22.8%
	Don’t know/Can’t remember	5.9%
Technology confidence	Very confident	32.0%
	Fairly confident	42.3%
	Not very confident	18.6%
	Not at all confident	7.2%
Technical problems	None	82.8%
	Minor	9.5%
	Major	3.3%
Need for help to join	No help needed	87.0%
	Needed assistance	9.5%
Satisfaction	Very satisfied	28.5%
	Satisfied	39.9%
	Neither satisfied nor dissatisfied	22.9%
	Dissatisfied	5.4%
	Very dissatisfied	3.3%
Future intention	Likely to continue	69.2%

### Factors associated with virtual consultation use

In unadjusted logistic regression analyses, ever having used a virtual consultation (VC) was associated with several sociodemographic characteristics. Ever-use of virtual consultations was most strongly associated with younger age, with participants aged 18–44 years significantly more likely to use VCs than those aged 65 years or older (OR 6.10, 95% CI 2.58–14.39; *p* < 0.001).

Use was also more likely with ethnic minority background (OR 1.72, 95% CI 1.04–2.84; *p* = 0.034), higher educational attainment (undergraduate degree: OR 2.19, 95% CI 1.31–3.68; *p* = 0.003), higher social grade (ABC1: OR 1.70, 95% CI 1.08–2.68; *p* = 0.021), and greater digital health literacy (eHEALS, per 1-point increase: OR 1.71, 95% CI 1.17–2.50; *p* = 0.006). Employment showed a borderline association (OR 1.56, 95% CI 0.99–2.48; *p* = 0.057), whereas gender, postgraduate education, and multimorbidity were not significantly associated with use (Figure 2).

**Figure 2:**
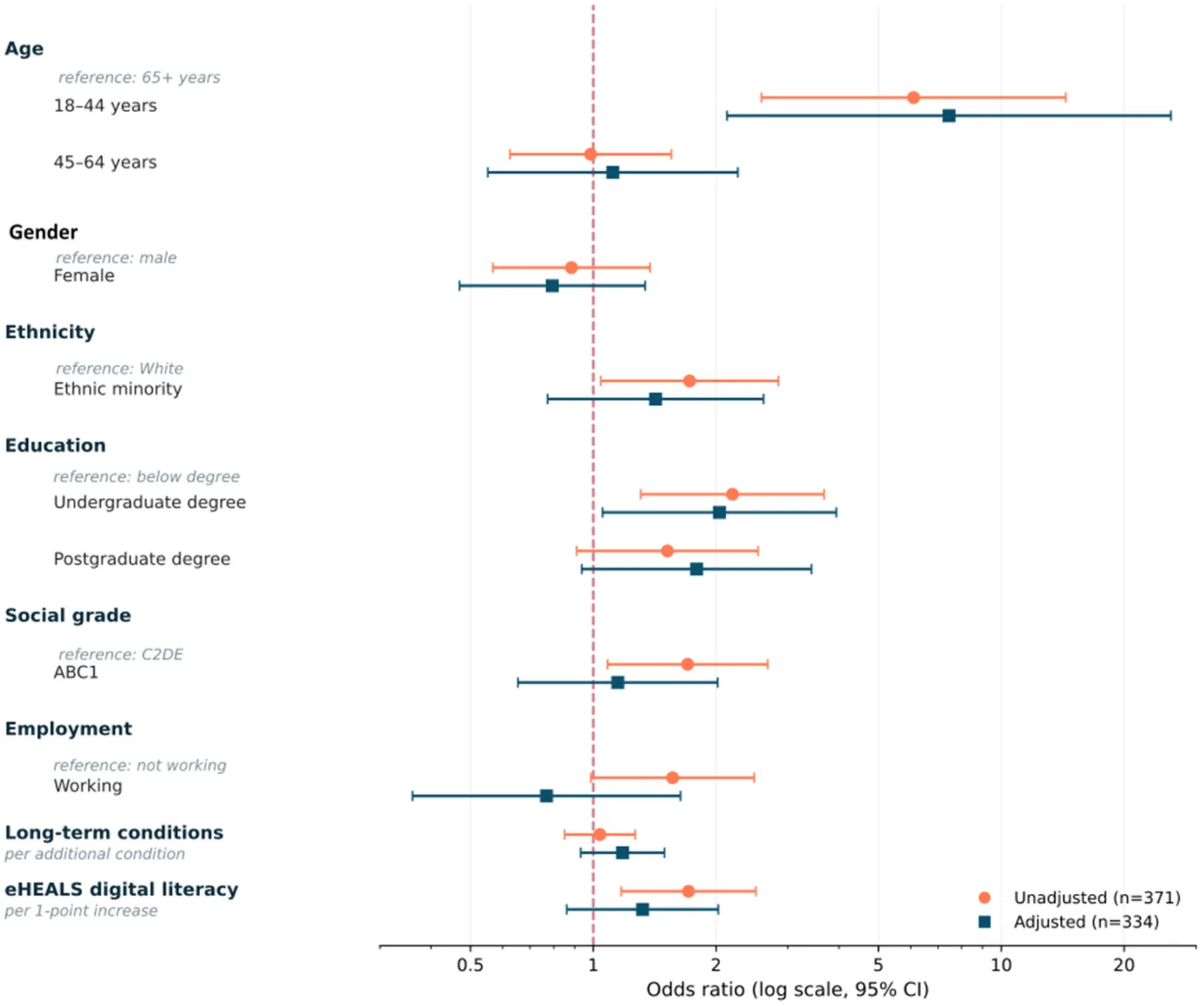
Determinants of ever-use of virtual consultations for type 2 diabetes. Weighted logistic regression: unadjusted (orange circles) and adjusted (navy squares) odds ratios with 95% CI on a log scale. Reference categories are shown in italics for categorical predictors; continuous predictors are expressed per unit increase. OR > 1 indicates higher odds of ever-use. Estimates are survey-weighted; adjusted model *n* = 334, pseudo-*R*^2^ = 0.087. eHEALS, eHealth Literacy Scale; CI, confidence interval.

In the multivariable model (*n* = 334; AIC = 398.2), only younger age and undergraduate educational attainment remained independently associated with VC use. Participants aged 18–44 years had significantly higher odds of VC use than those aged 65 years or older (OR 7.76, 95% CI 2.22–27.16; *p* = 0.001), while undergraduate education remained independently associated with VC use (OR 2.05, 95% CI 1.06–3.96; *p* = 0.033). The association for postgraduate education was attenuated and no longer significant after adjustment (OR 1.80, 95% CI 0.94–3.42; *p* = 0.075). Associations with ethnicity, social grade, employment status, and eHEALS scores were reduced and no longer statistically significant after adjustment. This pattern suggests considerable overlap between these characteristics, with younger, more highly educated, digitally literate, and economically active individuals tending to cluster within the same groups.

A sensitivity analysis excluding employment status, which was strongly correlated with age, yielded similar findings. The association between younger age and VC use remained robust and was slightly attenuated (OR 5.96, 95% CI 2.00–17.72; *p* = 0.001), supporting the stability of the main results.

### Factors influencing actual use/intention of using virtual consultations

The UTAUT2 model includes seven core constructs that explain why people adopt and use technology. Across the whole sample, agreement that each construct mattered varied widely. Effort Expectancy attracted the highest agreement, with 60.6–62.4% agreeing or strongly agreeing. Views on the Facilitating Conditions were mixed: most participants had the necessary technology (72.1%), but far fewer could get technical help (33.1%). Social Influence had the lowest agreement (12.7–25.3%). An overview of UTAUT2 mean scores, for each construct, is provided in Figure 3.

**Figure 3:**
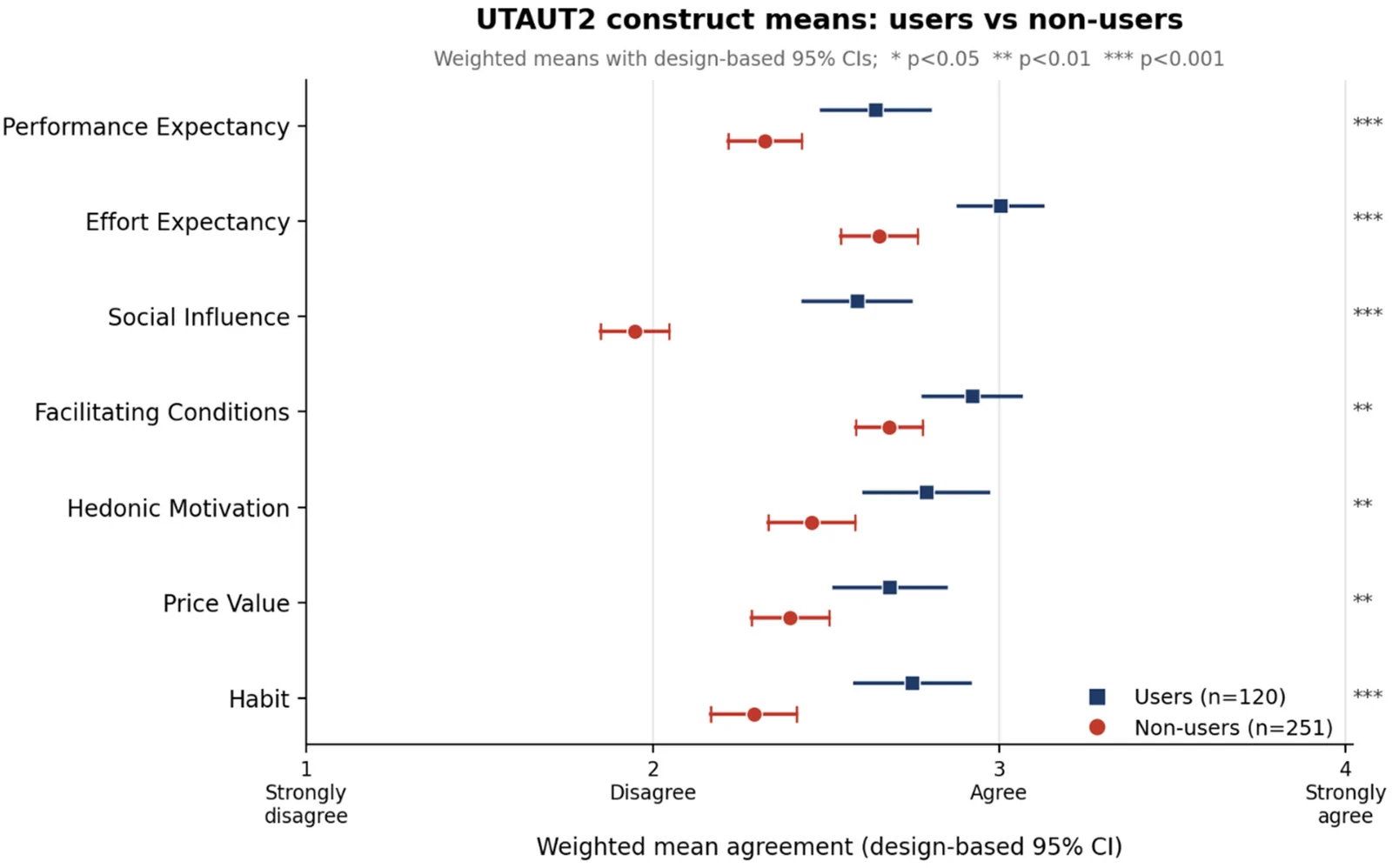
The unified theory of acceptance and Use of technology 2 (UTAUT2) means for users (navy squares) and non-users (red circles). Weighted mean agreement scores for users (*n* = 120), non-users (*n* = 251) across the seven UTAUT2 constructs shown as points with design-based 95% confidence intervals (users = navy squares, non-users = red circles), measured on a 4-point Likert scale (1 = strongly disagree, 4 = strongly agree; higher scores indicate stronger agreement). Users scored higher than non-users on every construct. Asterisks denote the statistical significance of the difference between users and non-users (*p < 0.05, **p < 0.01, ***p < 0.001).

Descriptive statistics stratified by user status showed that all seven UTAUT2 constructs were rated higher among users than non-users (Supplementary Table S5). Social Influence and Habit showed the largest differences between users and non-users (mean differences of 0.64 and 0.46, respectively).

Among users, “Effort Expectancy” was the strongly endorsed construct, with the highest mean score of any construct (3.00) and consistently high levels of agreement (∼60%–62%). This was closely followed by “Facilitating Conditions” (mean 2.92), indicating that ease of use and perceived support infrastructure were key enablers of virtual consultation.

In contrast, among non-users, “Facilitating Conditions” was the strongly endorsed construct (mean 2.68) and “Social Influence” was the weakest construct (mean 1.95). Non-users rated social and behavioural factors with lower importance, including low/modest agreement rates for the importance of “Habit” (mean 2.29) and “Performance Expectancy” (mean 2.32).

### Perceived impact on quality of care

Across the whole sample, the perceived positive impact of virtual consultations differed markedly between quality domains. The proportion agreeing that VCs had, or would have, a positive impact was highest for efficiency (62.4%), followed by timeliness (47.6%), safety (41.7%), equity (40.2%), effectiveness (36.9%), and patient-centredness (34.5%). A detailed overview is provided in Supplementary Table S4.

Across all five quality domains, perceived positive impact was consistently higher among users than non-users (all *p* < 0.01; Supplementary Table S4). Taking each domain in the same order: patient-centredness (users 44.5% vs. non-users 29.9%; *p* = 0.016), effectiveness (59.3% vs. 26.7%; *p* < 0.001), efficiency (72.7% vs. 57.7%; *p* = 0.0005), safety (62.5% vs. 32.2%; *p* < 0.001), and timeliness (57.3% vs. 43.2%; t = 3.21, *p* = 0.001). The largest differences between users and non-users were observed for effectiveness and safety. For equity, 40.2% agreed that people from different backgrounds can benefit equally from virtual consultations, whereas 24.1% disagreed and 35.7% were unsure. The full distribution of responses by domain is shown in Figure 4. These user versus non-user comparisons were repeated using design-based tests on weighted means (Supplementary Table S4): all five parallel domains remained significant and survived Benjamini–Hochberg correction, whereas the equity difference was not significant (design-based *p* = 0.45).

**Figure 4:**
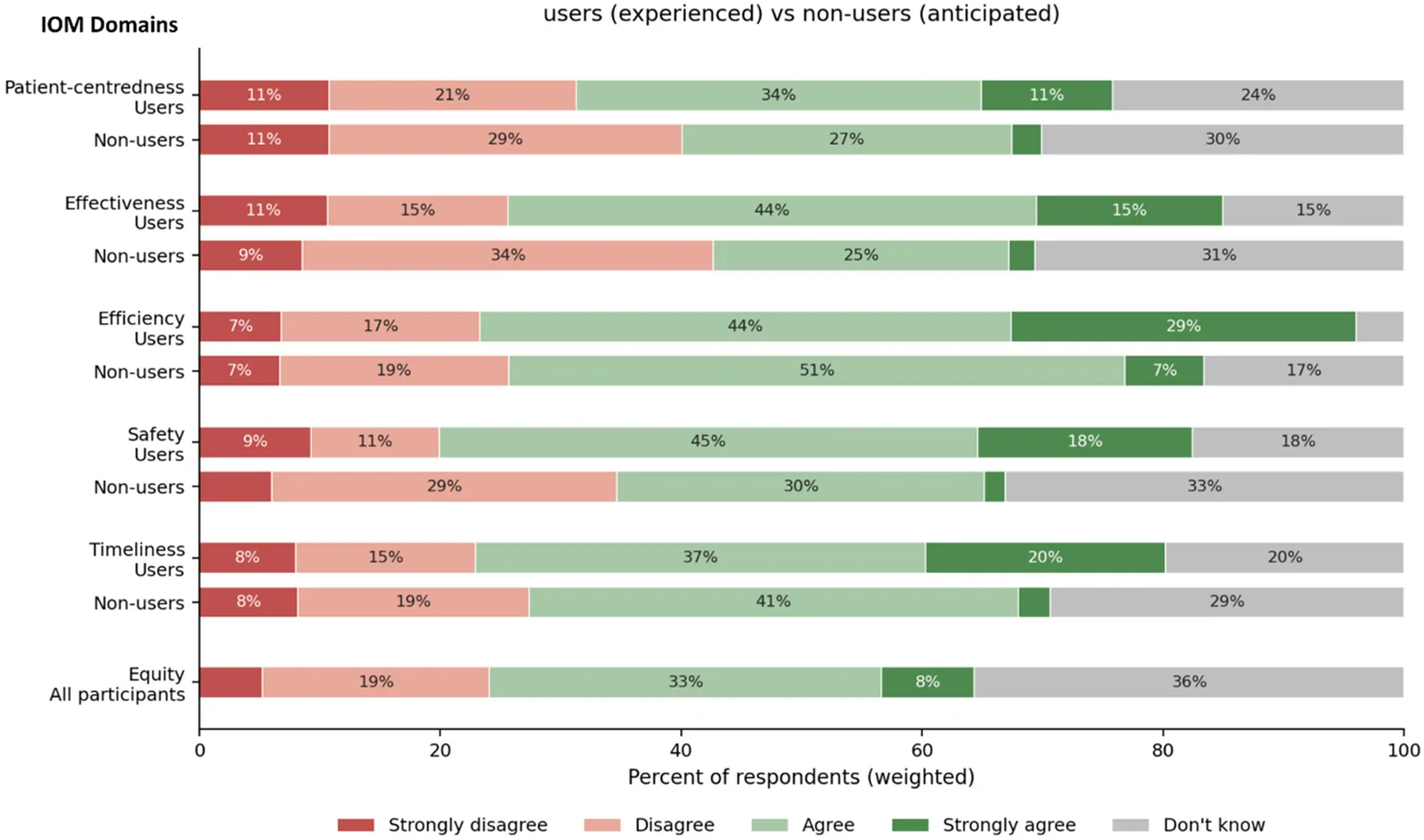
Perceived impact of virtual consultations across the six IOM quality-of-care domains. The five parallel domains show users’ (experienced) versus non-users’ (anticipated) ratings; the equity item was asked of all participants.

## Discussion

### Principal findings

This national survey addressed three questions: how virtual consultations are used by people with T2D and what predicts use; what factors shape their actual or intended use; and how patients perceive their impact on care quality.

First on pattern of use, roughly one in three respondents (31.0%) had used a virtual consultation for their T2D, and use was predominantly by telephone (91.3%) rather than video, and generally infrequently. Use was strongly socially patterned: only younger age and undergraduate education remained independent predictors, reflecting the overlap between these comparatively advantaged groups. Digital health literacy, rather than any demographic characteristic, was the only independent predictor of satisfaction among users.

For factors influencing use and intention, adoption beliefs (UTAUT2) varied across the sample, with effort expectancy attracting the most agreement and social influence the least. Users scored higher than non-users on agreement with every construct, with the largest differences being for social influence and habit.

Perceptions of impacts of virtual consultation on quality were uneven across IOM domains: efficiency was rated most favourably and patient-centredness and effectiveness least. Users rated the five parallel domains more positively than non-users anticipated, most for effectiveness and safety, whereas the equity item (asked of all respondents) did not differ significantly; and non-users gave far more “don't know” responses. Only a minority, even among users, agreed that people from different backgrounds benefit equally.

### Comparison with existing literature

Our finding that telephone, not video, dominated real-world use, being used by more than nine in ten users, echoes UK primary care studies showing that the pandemic-era shift to remote care was carried overwhelmingly by telephone, with video remaining rare (3). It also mirrors national NHS data: around a third of general practice appointments in England were delivered remotely by mid-2025 (33.3% in June 2025, up from 30.9% a year earlier), the great majority by telephone (about 25% of all appointments) rather than online or video (about 8%) (18). The conceptual unit differs, our 31.0% reflects patients who had ever used a virtual consultation, not a share of appointments, but both identify telephone as the dominant remote modality and video as marginal.

The concentration of use among younger, more educated, higher-social-grade and more digitally literate individuals mirrors the well-documented digital divide in remote care use (7, 19), and overall patient satisfaction with virtual care is generally comparable to in-person care, consistent with the wider virtual care literature (20, 21).

Our unadjusted finding that ethnic-minority respondents were more likely to have used virtual consultations (OR 1.72, 95% CI 1.04–2.84) runs counter to Alfarwan et al. (2025), who reported lower telemedicine use among Black individuals with type 2 diabetes in primary care. The association attenuated to non-significance after adjustment (OR 1.41, 95% CI 0.80–2.48), and our data contain only a binary White versus ethnic-minority classification, so the minority group (*n* = 120) pools populations with very different profiles and cannot be disaggregated. Whether this reflects a genuine UK contrast or an artefact of pooling therefore remains uncertain and merits dedicated study.

Our adoption findings align with technology-acceptance theory: effort expectancy (ease of use) was the most strongly endorsed construct, consistent with UTAUT2 evidence that perceived ease of use and facilitating conditions are central drivers of health-technology adoption (14). The relatively weaker role of social influence may suggest that, for an established clinical service, peer and provider encouragement matter less than personal capability and convenience (22, 23). Digital health literacy, rather than demographic characteristics, independently predicted satisfaction further suggests that capability shapes how positively virtual care is experienced (13, 24).

Perceptions of quality were more mixed. The comparatively favourable ratings for efficiency, alongside weaker ratings for patient-centredness and effectiveness, echo concerns that remote care can improve convenience while making relational, person-centred care harder to deliver (25). The equity finding that only a minority, even among users, believed all groups benefit equally reinforces calls to treat equity as an explicit quality domain in the design and evaluation of virtual care, rather than assuming that expanded access is inherently equitable (26, 27).

A further observation was the consistent gap between users’ experience and non-users’ expectations across every adoption construct, reinforced by the higher “don't know” rate among non-users (28). This is consistent with technology-acceptance theory, in which direct experience shapes performance expectancy, effort expectancy and habit (14), and implies that the adoption gap we observed may be partly addressable through low-stakes, well-supported first experiences. Importantly, however, modality and frequency of use are not determined by patient preference alone: in UK primary care the available mode is largely set by what each practice routinely offers, so observed patterns reflect local service provision as well as patient acceptance (3, 29).

### Strengths and limitations

This is one of the first UK national surveys to examine simultaneously factors such as patterns of virtual consultation use, determinants of adoption using UTAUT2 and patient perceptions across the six Institute of Medicine quality domains in people with T2D. Key strengths include a national sample drawn from a large research panel, the application of survey weights calibrated to UK population characteristics, the use of established instruments (eHEALS and UTAUT2), administered in an adapted four-point response format that showed internal consistency that was high for eHEALS, effort expectancy and performance expectancy but low for social influence and facilitating conditions (Cronbach's *α*: eHEALS 0.95, effort expectancy 0.84, performance expectancy 0.80, price value 0.81, social influence 0.66, facilitating conditions 0.62; hedonic motivation and habit were single items), and explicit grounding in the IOM quality framework. The parallel “experienced” versus “anticipated” design for users and non-users allowed reality to be compared directly against expectation.

This study has several limitations. The cross-sectional design precludes causal inference; in particular, the direction of the association between technology confidence and satisfaction cannot be established. Diagnosis of T2D and use of VCs were self-reported and not clinically verified, and patient-perceived quality and satisfaction were not linked to clinical outcomes such as HbA1c control. Reliance on an online research panel is likely to under-represent the most digitally excluded individuals, the very group of greatest equity concern. The relatively small number of users (*n* = 120) limited statistical power for adjusted and subgroup analyses and widened confidence intervals, as seen in the wide interval around the age effect. Income had substantial missingness (20.6%). Finally, neither the modality nor the frequency of virtual consultations is wholly patient-determined; in UK primary care both are strongly shaped by what individual practices offer, so our findings on use reflect a combination of patient characteristics and local service provision rather than patient preference alone.

### Implications for practice

As use and satisfaction were patterned by age, education, and digital health literacy rather than by the limited clinical need measure we were able to model, services should avoid assumptions about patients’ digital readiness and instead tailor the consultation modality to individual needs and circumstances. Practices should proactively identify patients at risk of digital exclusion, keep face-to-face and assisted options readily available, and provide additional support (set-up help, clear instructions, telephone alternatives) where needed. Because experience is far more positive than expectation, services should convert willing non-users through supported, low-stakes first consultations and clear expectation-setting rather than assuming reluctance is fixed; and because digital health literacy predicted satisfaction, strengthening patients’ capability to use virtual care is likely to improve how it is experienced.

### Implications for policy

One of the key findings from the study was that over 90% of virtual consultations were by telephone, which require much less digital capability than video. The strong endorsement of effort expectancy and facilitating conditions suggests that equitable scale-up depends on perceived clinical benefit, the ease of using digital tools and the availability of supporting resources; policymakers should therefore invest in usability, technical support and digital infrastructure. To protect equity, services should maintain multiple access routes (including non-digital ones), invest in digital-inclusion programmes, and monitor equity explicitly as a quality indicator rather than assuming that expanded access is inherently equitable. The uneven perceived benefits across domains reinforce that virtual consultations should complement rather than replace face-to-face care, within flexible, patient-tailored models that match modality to patients’ needs, preferences and context, designed and evaluated against person-centred and equity criteria, not efficiency alone.

These priorities map directly onto NHS England's digital-inclusion framework—access to devices and data; accessibility and ease of use; skills and capability; beliefs and trust; and leadership and partnerships—alongside maintained non-digital access, which offers a practical structure for delivering virtual diabetes care equitably. For a Health Technology Implementation audience, our measures speak to acceptability; adoption, reach, fidelity and sustainability were not assessed and remain priorities for evaluation.

## Conclusion

Virtual consultations should not be seen as a universal replacement for face-to-face care but as one component of a patient-centred, hybrid model of diabetes management. Their long-term success will depend on maintaining equitable access while ensuring that consultation modality is matched to patients’ clinical needs, preferences, and digital capability. Equitable and sustained uptake of virtual consultations requires attention to both digital inclusion and usability. Widening its benefits will depend on supported first experiences, building patients’ digital capability, sustaining non-digital access routes, and treating equity and person-centredness as core quality goals. Further research, ideally linking patient-reported perceptions to routinely collected clinical outcomes, is needed to establish whether these perceived impacts translate into measurable differences in care quality and to identify where virtual and face-to-face care are best deployed.

## Data Availability

The data supporting the conclusions of this article are available from the corresponding author on reasonable request, subject to the data-sharing terms of the YouGov panel and participant consent.
