# Supplementary material for: Patient perspectives on virtual consultations for type 2 diabetes in the United Kingdom: use, associated factors, and perceived quality of care
Source: Front Digit Health. 2026 Oct 12;8:1921014. doi: 10.3389/fdgth.2026.1921014 (PMC13696445; doi:10.3389/fdgth.2026.1921014)
Supplement: Supplementary file 1 [file Supplementaryfile1.docx]

# **Supplementary Material**

**Patient Perspectives on Virtual Consultations for Type 2 Diabetes in the United Kingdom: Use, Digital Adoption Determinants, and Perceived Impact on Quality of Care**

**Authors:** Reham Aldakhil^1^, Geva Greenfield^1^, Albandari Alharbi^1^, Ahmed Alboksmaty^2^, Benedict Hayhoe^1^, Azeem Majeed^1^, Ana Luisa Neves^1^

Supplementary Tables S1–S7 provide the survey structure (S1), objective-to-analysis mapping (S2), full regression results (S3), perceived quality-of-care impacts by user status (S4), UTAUT2 construct statistics (S5), the completed CHERRIES checklist (S6), and item- and group-specific missingness (S7).

**Table S1 Survey Development**

| Part | Questions | Content | Framework / Source | Routing |
| --- | --- | --- | --- | --- |
| I | Q1–Q9 | Demographics (Q1–Q4), clinical profile (Q5–Q8), digital health literacy (Q9) | eHEALS (Norman & Skinner, 2006) | All participants (age, gender, ethnicity, region, social grade from YouGov profiling) |
| Routing | Q10 | Ever used VC for T2D? (Yes / No / Prefer not to say) | Bespoke | All participants |
| II | Q11–Q17 | VC frequency, duration, modalities used, satisfaction, tech confidence, tech problems, help needed | Bespoke | VC Users only |
| III | Q18–Q29 | Technology adoption: Performance Expectancy (2), Effort Expectancy (2), Social Influence (2), Facilitating Conditions (2), Hedonic Motivation (1), Price Value (2), Habit (1) | UTAUT2 (Venkatesh et al., 2012) | All participants |
| IV | Q30–Q40 | Quality of care: Patient-centredness, Effectiveness, Efficiency, Safety, Timeliness, Equity (6 items) | IOM Six Domains of Healthcare Quality (IoM, 2001) | Q30–34: Users only, Q35–39: Non-users only, Q40: All |
| V | Q41–Q42 | Likelihood to continue (users) or try (non-users) VC | Bespoke | Q41: Users, Q42: Non-users |

*All Likert-type items used four-point agreement scales (strongly disagree to strongly agree) plus "Don't know". The eHEALS and UTAUT2 items were administered in this adapted four-point format rather than the original five-point (eHEALS) and five- to seven-point (UTAUT2) response scales. The satisfaction item used a five-point scale. Demographic variables were captured from YouGov's existing panel profiling data.*

**Table S2 Mapping of Study Objectives to Survey Questions and Analyses**

| Objective | Survey questions | Key variables | Analyses |
| --- | --- | --- | --- |
| 1. Use, experience & adoption of VCs , and how these differ across patient characteristics | Q10 (ever-use); Q11–Q13 (frequency, duration, modality , users); Q14–Q17 (satisfaction, technology confidence, technical problems, help needed , users); Q18–Q29 (UTAUT2 , all); Q9 (eHEALS) and Q1–Q8 + YouGov profiling (patient characteristics) | Ever-use of VC (binary outcome); usage frequency/duration/modality; satisfaction (high satisfaction, binary); technology confidence; technical problems; help needed; eHEALS digital health literacy; seven UTAUT2 construct scores; demographic & clinical characteristics (comparison groups/predictors) | Weighted descriptives; χ² tests of use by patient characteristics (Table 1); usage & experience profile (Table 2); weighted unadjusted & adjusted logistic regression of determinants of ever-use (Figure 2; Supplementary Table S3); logistic regression of satisfaction predictors; UTAUT2 construct means by user status (Figure 3; Supplementary Table S5); Cronbach's α for eHEALS and UTAUT2 |
| 2. Perceived quality of care across the six IOM domains | Q30–Q34 (users , experienced); Q35–Q39 (non-users , anticipated); Q40 (equity , all) | Perceptions across the six IOM domains (patient-centredness, effectiveness, efficiency, safety, timeliness, equity); experienced (users) vs anticipated (non-users) | Weighted descriptives (net agreement, means); users vs non-users comparisons (independent-samples t-tests) across the five comparable domains (Figure 4; Supplementary Table S4); equity item summarised for the whole sample |
| 3. Future intentions to use VCs | Q41 (continue , users); Q42 (try , non-users) | Intention to continue (current users); intention to try (non-users) | Weighted descriptives; group comparison of intentions between users and non-users |

**Table S3 Determinants of ever-use of virtual consultations: weighted unadjusted and adjusted logistic regression.**

| Variable | Unadjusted OR [95% CI] | p | Adjusted OR [95% CI] | p |
| --- | --- | --- | --- | --- |
| Age 18–44 (vs 65+) | 6.10 [2.58–14.39] | <0.001 | 7.76 [2.22–27.16] | 0.001 |
| Age 45–64 (vs 65+) | 0.98 [0.62–1.55] | 0.950 | 1.16 [0.57–2.34] | 0.681 |
| Female (vs male) | 0.88 [0.57–1.38] | 0.586 | 0.83 [0.50–1.40] | 0.489 |
| Ethnic minority (vs White) | 1.72 [1.04–2.84] | 0.034 | 1.41 [0.77–2.59] | 0.266 |
| Undergraduate (vs below degree | 2.19 [1.31–3.68] | 0.003 | 2.05 [1.06–3.96] | 0.033 |
| Postgraduate (vs below degree) | 1.52 [0.91–2.54] | 0.109 | 1.80 [0.94–3.42] | 0.075 |
| Social grade ABC1 (vs C2DE) | 1.70 [1.08–2.68] | 0.021 | 1.14 [0.65–1.99] | 0.655 |
| Working (vs not working) | 1.56 [0.99–2.48] | 0.057 | 0.72 [0.34–1.53] | 0.399 |
| Multimorbidity | 0.82 [0.47–1.41] | 0.469 | 1.20 [0.63–2.29] | 0.571 |
| eHEALS mean (per 1-point) | 1.71 [1.17–2.50] | 0.006 | 1.30 [0.85–2.00] | 0.228 |

Unadjusted model n = 371; adjusted model n = 334, AIC = 398.2, McFadden pseudo-R² = 0.087. Estimates are survey-weighted. OR, odds ratio; CI, confidence interval; eHEALS, eHealth Literacy Scale.

**Table S4 Patient-perceived impact of virtual consultations across the Institute of Medicine (IOM) quality-of-care domains.**

| Domain | Users net agree | Users mean | Non-users net agree | Non-users mean | t | p-value |
| --- | --- | --- | --- | --- | --- | --- |
| Patient-centredness | 44.5% | 2.59 | 29.9% | 2.31 | 2.62 | 0.009 |
| Effectiveness | 59.3% | 2.75 | 26.7% | 2.29 | 4.66 | <0.001 |
| Efficiency | 72.7% | 2.98 | 57.7% | 2.69 | 3.21 | 0.001 |
| Safety | 62.5% | 2.86 | 32.2% | 2.42 | 4.53 | <0.001 |
| Timeliness | 57.3% | 2.86 | 43.2% | 2.53 | 3.21 | 0.001 |
| Equity | 51.2% | 2.72 | 35.6% | 2.63 | 0.84 | 0.404 |

All figures are weighted. Net agree = "Agree" + "Strongly agree" as a proportion of the full group (including "Don't know"); means are on a 1–4 agreement scale excluding "Don't know"; group comparisons are independent-samples (pooled-variance) t-tests. Equity was asked of all participants; 40.2% of the full sample agreed overall.

**Table S5 Descriptive statistics for the seven UTAUT2 constructs, total sample and stratified by user status.**

| Construct | Items | Net Agree (%) | Total Mean | α | Users mean agreement | Non-users mean agreement | p-value |
| --- | --- | --- | --- | --- | --- | --- | --- |
| Performance Expectancy | 2 | 24.7–43.7 | 2.44 | 0.799 | 2.64 | 2.32 | 0.002 |
| Effort Expectancy | 2 | 60.6–62.4 | 2.77 | 0.838 | 3.00 | 2.65 | <0.001 |
| Social Influence | 2 | 12.7–25.3 | 2.15 | 0.664 | 2.59 | 1.95 | <0.001 |
| Facilitating Conditions | 2 | 33.1–72.1 | 2.76 | 0.623 | 2.92 | 2.68 | 0.011 |
| Hedonic Motivation | 1 | 48.0 | 2.59 | N/A | 2.79 | 2.46 | 0.004 |
| Price Value | 2 | 30.8–49.2 | 2.50 | 0.808 | 2.69 | 2.40 | 0.005 |
| Habit | 1 | 42.8 | 2.46 | N/A | 2.75 | 2.29 | <0.001 |

Mean agreement score per construct on the 1–4 scale (higher = stronger agreement). Cronbach's α is reported for the multi-item constructs; internal consistency was high for eHEALS (α = 0.95), effort expectancy (0.838) and performance expectancy (0.799), but low for social influence (0.664) and facilitating conditions (0.623). Hedonic motivation and habit were single items, for which α is not estimable. Construct scores for the low-α constructs should be interpreted with corresponding caution. Two facilitating-conditions items are reported separately in the main text (72.1% had the necessary technology; 33.1% could get technical help). Group comparisons use design-based tests.

**Table S6 CHERRIES checklist**

Checklist for Reporting Results of Internet E-Surveys (CHERRIES).

| Checklist item | CHERRIES recommendation (what to report) | How addressed in this study |
| --- | --- | --- |
| Design | | |
| Describe survey design | State the target population and sampling frame, and whether the sample is a convenience sample. | Cross-sectional online survey of UK adults (≥18 years) with self-reported type 2 diabetes (T2D), drawn from the YouGov UK research panel (~400,000 members), specifically the >9,600 panellists with a recorded T2D profile. Non-probability panel sample; survey weights applied to reflect UK population characteristics. |
| IRB (ethics) approval and informed consent process | | |
| Ethics / IRB approval | State whether the study was approved by a research ethics committee / IRB. | Approved by the Imperial College Research Ethics Committee (ICREC), reference number 8213600, on 29 January 2026, following review by the Research Governance and Integrity Team (RGIT) and the Head of Department. |
| Informed consent | Describe the informed consent process: were participants told the time required, what data were stored (where/how long), who the investigator was, and the study purpose? | All participants received an approved participant information sheet and provided informed consent (approved consent form, v1.2) before completing the survey. |
| Data protection | Describe safeguards used to protect any personal data collected or stored. | Data handling and storage were described in the approved participant information sheet ; demographic profiling is held by YouGov; responses were analysed by the research team in de-identified form. Personal data are retained in line with YouGov's privacy notice (held only as long as needed or required by law, or until erasure is requested) and are encrypted in transit (TLS) and at rest (AES-256). |
| Development and pre-testing | | |
| Development and testing | State how the instrument was developed and whether its usability and technical functionality were tested before fielding. | Questionnaire developed from validated instruments (eHEALS; UTAUT2) and the IOM quality-of-care domains. The instrument was piloted before launch, (Q17, Q18) were revised: they were reworded from asking only about the respondent's own experience to “based on what you've seen/heard about, or your personal experience…”, and were routed to apply only to relevant respondents (asked if Q7==1). |
| Open vs closed survey | State whether the survey was open to any site visitor or restricted to a known (e.g., password-protected) sample. | Closed survey: accessible only to invited YouGov panellists profiled with T2D. |
| Recruitment process and description of the sample having access to the questionnaire | | |
| Contact mode | State whether the initial contact with potential participants was made online. | Online: panel/email invitations sent to panellists profiled with T2D. |
| Advertising the survey | Describe how and where the survey was announced/advertised, including the wording, as this can shape self-selection. | Not publicly advertised; closed invitations issued by YouGov to eligible panellists. Invitations are managed automatically by YouGov's panel and sampling software, which sends a steady flow of non-specific survey invitations; on clicking through, a panellist is allocated to a survey matching their demographics. Consequently, the total number of panellists invited to this specific survey cannot be calculated. |
| Survey administration | | |
| Web / e-mail | State the type of e-survey and whether responses were captured automatically or entered manually. | Web-based survey hosted on the YouGov platform; responses captured automatically. |
| Context | Describe the panel/site in which the survey ran and how its character might pre-select or influence the sample. | YouGov maintains a general-purpose UK opinion/research panel; this sample was drawn from members with a recorded T2D profile. |
| Mandatory / voluntary | State whether completing the survey was voluntary. | Voluntary. |
| Incentives | State whether any incentives (monetary, prizes, or non-monetary) were offered. | Standard YouGov panel points, Standard YouGov panel reward points, awarded under the panel's scheme (points scale with survey length/complexity and are redeemable via the YouGov Wallet). |
| Time / date | Report the data-collection timeframe. | 30 January – 6 February 2026. |
| Randomization of items | State whether item or response-option order was randomised/alternated to reduce bias. | Yes, randomisation was applied wherever appropriate, e.g. for lists with no inherent order (such as non-time-series response options). |
| Adaptive questioning | State whether conditional/branching (adaptive) questions were used. | Yes: a routing question classified respondents as VC users or non-users and directed subsequent items accordingly. |
| Number of items | Report the number of questionnaire items (and items per page). | Instrument comprised eHEALS (8 items), UTAUT2 (12 items), 11 quality-domain items, plus demographic/clinical items. |
| Number of screens (pages) | Report over how many pages/screens the survey was distributed. | Each question was presented on its own page; some grid questions were dynamic, presenting one row per page. The exact number of pages therefore varied by respondent because of routing. |
| Completeness check | State whether completeness/consistency checks were applied and whether a non-response option was offered for each item. | “Don't know” / non-response options were available and later recoded to missing. |
| Review step | State whether respondents could review and change their answers (e.g., a Back button or review screen). | Back step, review screen step was available. |
| Response rates | | |
| Unique site visitor | If view/participation rates are reported, define how a unique visitor was determined (IP, cookies, or both). | Not applicable in the usual web-traffic sense: a closed panel sample identified by unique panellist accounts rather than open site traffic. |
| View rate | Ratio of unique survey visitors ÷ unique site visitors (2012 erratum). | Not applicable (closed panel; no open site traffic). |
| Participation rate | Ratio of those who agreed to participate ÷ unique first-survey-page visitors (2012 erratum). | Not calculable: because invitations are issued automatically and non-specifically by the panel software, the number invited to this survey is not available. |
| Completion rate | Ratio of those who finished the survey ÷ those who agreed to participate (2012 erratum). | 91.1%: 380 of 417 respondents who initiated the survey completed it. |
| Preventing multiple entries from the same individual | | |
| Cookies used | State whether cookies were used to give each computer a unique ID and to prevent duplicate entries. | Cookies form part of YouGov's multi-layered quality-assurance process. This includes a proprietary Response Quality Survey (RQS) with 20+ behavioural checks, continuously refined using AI/machine learning, to verify that panellists meet quality standards. |
| IP check | State whether IP addresses were used to detect duplicate entries, and how duplicates were handled. | IP checks are part of the same quality-assurance process. Third-party tools (e.g. Research Defender) flag risks such as location spoofing, TOR usage, or professional survey-taking; medium- or high-risk respondents are excluded from fieldwork. |
| Log file analysis | State whether any log-file techniques were used to identify multiple entries. | Because YouGov owns and manages its panel, respondent behaviour is monitored over time; survey-level controls include screening, consistency checks, completion-speed monitoring, and review of open-ended responses to remove low-quality or suspicious entries. |
| Registration | For closed surveys, describe how login prevented duplicate entries. | Closed survey: each panellist accessed the survey through a unique, authenticated panel account, preventing repeat submission. |
| Analysis | | |
| Handling of incomplete questionnaires | State whether only completed questionnaires were analysed, or whether early-terminated responses were also included. | Only completed responses were used; after quality screening, 7 of 380 were excluded for inconsistent answers or substantial missing data, giving the final analytic sample of n=373. |
| Atypical timestamp | State whether questionnaires submitted unusually quickly (speeders) were excluded. | Quality screening removed inconsistent responses; completion speeds are monitored as part of YouGov's standard quality controls. |
| Statistical correction | State whether weighting or other methods were used to adjust for a non-representative sample. | YouGov survey weights were applied to all descriptive and inferential analyses so that estimates reflect the UK adult T2D population rather than the unweighted panel. Weighting variables were gender, age, ethnicity, and social grade, with population targets sourced from the NHS National Diabetes Audit. The effective base was 1,421 for the nationally representative sample and 500 for the T2D sample; the design effect can in principle be derived from the effective and achieved base sizes but is not routinely provided by the panel provider. |

**Table S7 item/group 'don't know' (missing) rates**

| Scale / domain | Users 'don't know' % | Users n | Non-users 'don't know' % | Non-users n |
| --- | --- | --- | --- | --- |
| UTAUT2: Performance Expectancy | 17.7 | 120 | 32.1 | 251 |
| UTAUT2: Effort Expectancy | 6.8 | 120 | 16.9 | 251 |
| UTAUT2: Social Influence | 35.9 | 120 | 35.7 | 251 |
| UTAUT2: Facilitating Conditions | 17.7 | 120 | 27.2 | 251 |
| UTAUT2: Hedonic Motivation | 3.9 | 120 | 24.8 | 251 |
| UTAUT2: Price Value | 15.5 | 120 | 31.0 | 251 |
| UTAUT2: Habit | 7.5 | 120 | 24.3 | 251 |
| IOM: Patient-centredness | 24.2 | 120 | 30.1 | 251 |
| IOM: Effectiveness | 15.1 | 120 | 30.6 | 251 |
| IOM: Efficiency | 4.0 | 120 | 16.6 | 251 |
| IOM: Safety | 17.6 | 120 | 33.1 | 251 |
| IOM: Timeliness | 19.8 | 120 | 29.4 | 251 |
| IOM: Equity (all) | 22.9 | 120 | 40.9 | 251 |

'Don't know' rates (weighted) by group. For UTAUT2 multi-item constructs the value is the mean item rate. 'Don't know' is consistently more common among non-users, indicating responses are not missing at random; a sensitivity analysis recoding 'don't know' to the scale midpoint left all conclusions unchanged (12/13 comparisons significant after BH; equity non-significant, p=0.29). Demographic missingness: household income 20.6% (n=77), education 4.6% (n=17), long-term conditions 3.2% (n=12), employment 1.3% (n=5), self-rated management 0.8% (n=3).
